# Omega-3 Polyunsaturated Fatty Acids in Depression

**DOI:** 10.3390/ijms25168675

**Published:** 2024-08-08

**Authors:** Anna Serefko, Monika Elżbieta Jach, Marlena Pietraszuk, Małgorzata Świąder, Katarzyna Świąder, Aleksandra Szopa

**Affiliations:** 1 Department of Clinical Pharmacy and Pharmaceutical Care, Medical University of Lublin, Chodźki Street 7, 20-093 Lublin, Poland; aleksandra.szopa@umlub.pl; 2 Department of Molecular Biology, The John Paul II Catholic University of Lublin, Konstantynów Street 1I, 20-708 Lublin, Poland; monika.jach@kul.pl; 3 Student Scientific Club, Department of Clinical Pharmacy and Pharmaceutical Care, Medical University of Lublin, Chodźki Street 7, 20-093 Lublin, Poland; 59357@student.umlub.pl; 4 Student Scientific Club, Chair and Department of Applied and Social Pharmacy, Medical University of Lublin, Chodźki Street 1, 20-093 Lublin, Poland; 63595@student.umlub.pl; 5 Student Scientific Club, Department of Experimental and Clinical Pharmacology, Medical University of Lublin, 8b Jaczewskiego, 20-090 Lublin, Poland; 6 Chair and Department of Applied and Social Pharmacy, Medical University of Lublin, Chodźki Street 1, 20-093 Lublin, Poland; katarzyna.swiader@umlub.pl

**Keywords:** omega-3, polyunsaturated fatty acids, depression, alpha-linolenic acid, docosahexaenoic acid (DHA), eicosapentaenoic acid (EPA)

## Abstract

Omega-3 polyunsaturated fatty acids have received considerable attention in the field of mental health, in particular regarding the treatment of depression. This review presents an overview of current research on the role of omega-3 fatty acids in the prevention and treatment of depressive disorders. The existing body of evidence demonstrates that omega-3 fatty acids, in particular eicosapentaenoic acid (EPA) and docosahexaenoic acid (DHA), have antidepressant effects that can be attributed to their modulation of neuroinflammation, neurotransmitter function, and neuroplasticity. Nevertheless, clinical trials of omega-3 supplementation have yielded inconsistent results. Some studies have demonstrated significant reductions in depressive symptoms following omega-3 treatment, whereas others have shown minimal to no beneficial impact. A range of factors, encompassing dosage, the ratio of EPA to DHA, and baseline nutritional status, have been identified as having a potential impact on the noted results. Furthermore, it has been suggested that omega-3 fatty acids may act as an adjunctive treatment for those undergoing antidepressant treatment. Notwithstanding these encouraging findings, discrepancies in study designs and variability in individual responses underscore the necessity of further research in order to establish uniform, standardized guidelines for the use of omega-3 fatty acids in the management of depressive disorders.

## 1. Introduction

Major depression disorder is a worldwide disease that affects about 300 million people, both children and adults and females and males. Although its pathogenesis has not been fully described, there are several factors that contribute to the development of depression, including biologic (brain chemistry, genetics, and epigenetics), medical (chronic conditions, and medications), and psychosocial (stressful events, personality traits, and poor nutrition) [1,2]. According to *The Diagnostic and Statistical Manual of Mental Disorders, Fifth Edition, Text Revision (DSM-5-TR)* by the American Psychiatric Association [3], the disease is diagnosed in patients who present at least five specific symptoms of depression: (A) depressed mood, (B) markedly diminished interest or pleasure in activities, (C) significant unintentional weight loss or weight gain, (D) insomnia or hypersomnia, (E) psychomotor agitation or slowing, (F) fatigue or loss of energy, (G) feelings of worthlessness or excessive/inappropriate guilt, (H) diminished ability to think or concentrate or indecisiveness, or (I) recurrent thoughts of death or suicidal ideation. They should last nearly every day for 14 days. Furthermore, there are several other conditions needed to diagnose depression: (1) amongst the above-mentioned symptoms should be either depressed mood or markedly diminished interest or pleasure in activities; (2) the symptoms should cause clinically significant distress or impairment in social, occupational, or other important areas of functioning; (3) the symptoms are not attributable to a substance or other medical condition (including schizophrenia or other psychotic disorder), and the patient has never experienced a manic or hypomanic episode [3]. Unfortunately, most people who are depressed are undertreated or untreated [4], whereas treated patients often experience limitations of the therapy [5,6,7,8]. The keystones of depression treatment are still drugs that target the monoaminergic system, such as selective serotonin reuptake inhibitors (SSRIs), serotonin-noradrenaline reuptake inhibitors (SNRIs), noradrenaline and specific serotonergic antidepressants (NASSAs), serotonin antagonists and reuptake inhibitors (SARIs), or monoamine oxidase inhibitors (MAOIs) and tricyclic antidepressants (TCAs), which are used less often [9]. Apart from that, atypical (multimodal) antidepressants that act as dopamine and noradrenaline reuptake inhibitors (i.e., bupropion) or interact with serotonin, noradrenaline, or melatonin receptors (i.e., vortioxetine and agomelatine) are used. Quite recently, newer antidepressant drugs, including esketamine (a glutamatergic antagonist), brexanolone (a GABAA positive allosteric modulator), and combinations of bupropion and dextromethorphan (a low-affinity uncompetitive antagonist of NMDA receptors and sigma-1 receptor agonist) or fluoxetine hydrochloride and olanzapine, have been introduced into the pharmaceutical market [5,6]. But even though the pharmacotherapy of depression has been changing throughout recent decades, there are a lot of problems with available mood-improving drugs, including their side effects (i.e., headache, nausea, insomnia, gastrointestinal problems, sexual dysfunctions, weight gain), delayed onset of their action (several weeks) [5,6], and recurrence of the disease [10]. According to the Global Health Data Exchange [11], ca. 3.8% of the population suffers from depression, with 5% of adults (6% of women and 4% of men) and 5.7% of adults over 60 years old. About one-third of patients with depression have so-called treatment-resistant depression, i.e., when the patient does not respond to two or more different drugs that have been taken according to the approved treatment schedule (time and dosage) [6].

Problems with the management of depression with pharmacotherapy and/or psychotherapy have resulted in a growing interest in new non-pharmacological approaches, including the nutritional one [12]. Emerging evidence suggests that dietary factors may be related to mental health [13,14]. The Mediterranean diet, DASH (Dietary Approaches to Stop Hypertension) diet, MIND (Mediterranean-DASH Intervention for Neurodegenerative Delay) diet, healthy Nordic diet, and Okinawan diet are known for their beneficial effects on cognitive functions [15,16]. Fish and seafood, beans and legumes, vegetables, including leafy greens, olive oil, or nuts can be referred to as “brain food” [13]. Furthermore, Micek and colleagues [14] drew attention to polyphenol-rich beverages (e.g., coffee, tea, and red wine), the consumption of which can be associated with a lower rate of depressive symptoms and stress, whereas Lefevre-Arbogast et al. [17] highlighted the significance of prebiotic food for the healthy gut-brain axis. Among the proposed nutritional interventions in the management of depression, the supplementation of tryptophan, S-adenosyl-L-methionine, folic acid, vitamin D, zinc, or omega-3 polyunsaturated fatty acids is also mentioned [18,19].

Results of multiple preclinical studies suggest that insufficient intake of omega-3 polyunsaturated fatty acids may result in the development of depressive-like behavior in laboratory animals [20,21]. Based on observations of eating habits in the human population, it has been assumed that a Western diet with little fish consumption may increase the risk of depression, suicidal thoughts, anxiety, or seasonal affective disorder [22]. Several authors also detected that patients with depression may have lower levels of omega-3 polyunsaturated fatty acids (eicosapentaenoic acid—EPA and docosahexaenoic acid—DHA) both in the brain (post-mortemanalyses) and plasma [23,24,25,26]. Such an association has not been observed for omega-6 polyunsaturated fatty acids [24], though according to the recent meta-analysis by Wang and others [27], based on 12 cohort trials, a high ratio of omega-6 to omega-3 fatty acids is positively correlated with clinical symptoms of depression, and a low ratio of dietary-derived omega-6/omega-3 fatty acids may help to prevent depression. These observations were in compliance with findings by da Silva Sabião and others [28], who reported that a higher consumption of omega-6 fatty acids as well as a higher ratio of polyunsaturated to monounsaturated and saturated fatty acids were positively associated with the prevalence of depression. Furthermore, low levels of EPA in erythrocytes were correlated with the severity of depression [29] and suicide attempts [30]. Van der Burg and colleagues [31] demonstrated that EPA and DHA concentrations in red cell membranes were elevated in response to omega-3 supplementation, and they may be regarded as response biomarkers when treating depression since their levels were also correlated with attenuation of depressive symptoms. Based on the outcomes of 30 studies, Antao et al. [32] concluded that a low omega-3 index (i.e., the sum of EPA and DHA in red blood cells) is a risk factor for the development of several psychiatric diseases, including depression. As a risk threshold for the western population, they proposed an omega-3 index of 4–5% in major depression and 5% in postpartum depression.

Regarding the above-mentioned information, the main objective of our review article is to summarize the existing knowledge on the influence of omega-3 fatty acids supplementation on the prevention and treatment of depression in various populations (i.e., children, adults, elderly people, pregnant and breastfeeding women, as well as menopausal women). Furthermore, we have tried to explain the differences between study findings and present some ideas for further research in order to investigate the relationship between omega-3 fatty acid status and the development and/or remission of depression.

## 2. Omega-3 Polyunsaturated Fatty Acids

Omega-3 fatty acids (n-3 fatty acids or ω-3 fatty acids) are a type of polyunsaturated fatty acid. They are classified as polyunsaturated because they have multiple double bonds in their structure. The term “omega-3” signifies that a carbon–carbon double bond is located at the third carbon atom from the methyl (-CH_3_) end of the molecule, indicating its position in the omega end [33,34,35]. There are three main types of omega-3 fatty acids that are important in human nutrition, i.e., alpha-linolenic acid (ALA), EPA, and DHA [34,36]. ALA belongs to short-chain omega-3 fatty acids with 18 carbons and three double bonds (18:3, n-3). In turn, EPA and DHA have been classified as long-chain omega-3 fatty acids with 20 carbons and five double bonds (20:5, n-3) and 22 carbons and six double bonds (22:6, n-3), respectively [37] (Table 1).

The human body is incapable of creating carbon–carbon double bonds before the ninth carbon counting from the CH_3_- end of a fatty acid chain [38]. Unlike other fats, omega-3 fatty acids cannot be produced within the human body and must be obtained through diet. Therefore, they are called essential fatty acids [39,40]. While ALA can be metabolized into EPA and subsequently DHA, this conversion process is highly inefficient (efficiency < 15%). Thus, the most effective method to elevate these fatty acids in the body is by directly consuming EPA and DHA through food and/or dietary supplements [41].

**Table 1:** Omega-3 fatty acids chemical characterization.

Omega-3 Fatty Acid Name	Molecular Formula	Chemical Structure	Ref.
Alpha-linolenic acid (ALA)	C_18_H_30_O_2_	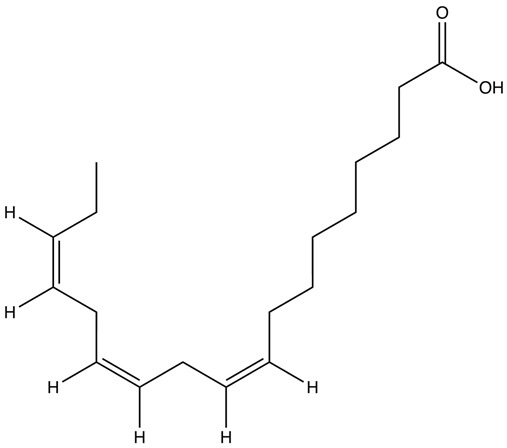	[42]
Eicosapentaenoic acid (EPA)	C_20_H_30_O_2_	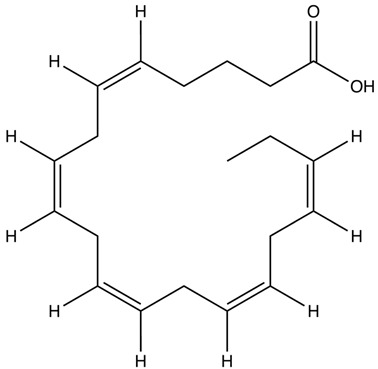	[43]
Docosahexaenoic acid (DHA)	C_22_H_32_O_2_	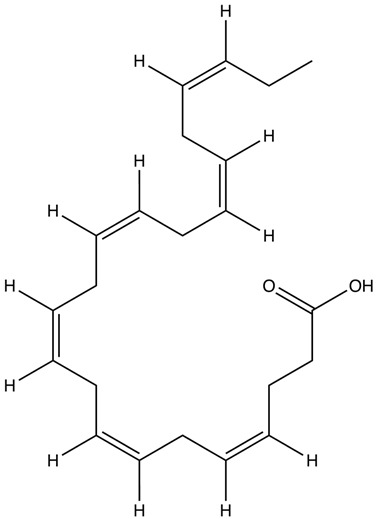	[44]

The content of ALA, EPA, and DHA varies across different food items. ALA is predominantly found in plant-based sources such as flaxseeds, chia seeds, and walnuts [45]. EPA is derived from ALA (though the conversion rate in the body is low) or consumed directly from marine sources, such as fatty fish (salmon, mackerel, and sardines) and algae. Like EPA, DHA can be synthesized from ALA in limited amounts or obtained directly from marine sources, including fatty fish, such as salmon, mackerel, and sardines [40,46] (Table 2).

Deficiency of essential fatty acids, including EPA, may manifest as excessive thirst with frequent urination or dermatological problems (dry skin, dry hair, brittle nails, dandruff, or small skin bumps) [48]. Over the years, various health and nutrition organizations worldwide have established guidelines to ensure optimal intake of fatty acids for health benefits. In 1989, during the NATO Workshop on Omega-3 and Omega-6 Fatty Acids, recommendations for the daily intake of EPA and DHA for the general adult population at the level of 300–400 mg were developed [49]. Afterward, the European Scientific Committee on Food settled the population reference intakes of polyunsaturated fatty acids amounting to 2.5% of total daily energy, of which, 2% should come from omega-6 and 0.5% from omega-3 fatty acids. This translates to a daily intake of around 5 + 1 g for women and 6.4 + 1.6 g for men [50]. Expanding on these guidelines, the International Society for the Study of Fatty Acids and Lipids (ISSFAL) in 2003 advocated for at least 500 mg/day of combined EPA and DHA, highlighting their importance for cardiovascular health [51]. Additionally, in 2007 the ISSFAL recommended that women who are pregnant/lactating should be given DHA at a dose of 200 mg/day [52]. The European Food Safety Authority (EFSA) further detailed that adults should consume 2 g/day of ALA and 250 mg/day of EPA and DHA, alongside a 10 g/day intake of linoleic acid (LA), i.e., omega-6 fatty acid [53]. Also, the World Health Organization (WHO) published its recommendations for omega-3 fatty acids; according to the document from 2002, for the general adult population, 1–2% of energy/day should come from the intake of omega-3 fatty acids [54]. In 2008, guidelines of three global organizations were published, i.e., the Food and Agriculture Organization of the United Nations (FAO) [55], World Association of Perinatal Medicine [56], and World Gastroenterology Organization [57]. The two first mentioned issued recommendations for pregnant and lactating women, i.e., 0.3 g/day of EPA + DHA (with a DHA amount of 0.2 g/day) [55] and 200 mg of DHA/day [56], respectively. Moreover, FAO provided guidelines for infants and children, according to which an adequate intake of DHA for 0–6-month-old babies should be 0.1–0.18% of energy, and for 6–24-month-old children—10–12 mg/kg per day. An adequate intake of EPA + DHA for 2–4-year-old children should be 100–150 mg/day, for 4–6-year-old children—150–200 mg/day, and for 6–10-year-old children—200–250 mg/day [55]. In the case of infants, when breastfeeding is not possible, the World Association of Perinatal Medicine recommends that omega-3 acids should constitute 0.2–0.5% by weight of total daily fat intake [56]. Additionally, most European countries, as well as Japan, the United States, Canada, Australia and many others, have recommendations regarding the necessary intake/supplementation of ALA, EPA, and DHA for the population of their country, taking into account age groups, gender, and special circumstances (e.g., pregnancy and lactation).

## 3. Potential Mechanisms of Action of Omega-3 Polyunsaturated Fatty Acids in Depression

Throughout the years, several theories related to the pathophysiology of depression have been developed. Among them, the monoamine theory is the most well-known; it states that deficiencies in monoamine neurotransmitters (serotonin, dopamine, and norepinephrine) are responsible for the development of depression. Linked to the monoamine hypothesis is the receptor hypothesis, according to which changes in the distribution, expression, or functioning of multiple receptors in the central nervous system, such as serotonin (5-hydroxytryptamine, 5-HT), dopamine, N-methyl-D-aspartate (NMDA), α-amino-3-hydroxy-5-methyl-4-isoxazolepropionic acid (AMPA), γ-aminobutyric acid (GABA), or glucocorticoid ones, may play a significant role in depression. Another one is the hypothalamic–pituitary–adrenal (HPA) axis hyperactivity hypothesis, which assumes that stressful events may lead to episodes of depression. An elevated activity of corticotropin-releasing hormone (CRH) occurs as a result of chronic stress, which in turn stimulates the production of pituitary adrenal corticotropic hormone (ACTH) and, as a consequence, glucocorticoids in the adrenal cortex. Higher levels of glucocorticoids may be damaging to neurons in the brain. The HPA axis hyperactivity hypothesis relates to the neurotrophic factor hypothesis and the neuroplasticity/neurogenesis hypothesis. In a situation when cortisol levels become very high and the brain is not able to control the excessive production of cortisol, decreased concentrations of the brain-derived neurotrophic factor (BDNF) are detected [1,58]. BDNF belongs to the neurotrophin family of growth factors, and it is synthesized in the endoplasmic reticulum. BDNF protein and mRNA can be found in most brain areas, including the ones important in the pathogenesis of depression, such as the olfactory bulb, cortex, hippocampus, forebrain, and hypothalamus [59]. This growth factor influences neuronal growth, differentiation, proliferation, migration and survival, synaptic plasticity, and release of neurotransmitters, as well as long-term potentiation [60]; therefore, people with neurodegenerative disorders (e.g., Parkinson’s disease, multiple sclerosis, or Huntington’s disease) present lower levels of BDNF [59]. Similarly, in patients suffering from depression or in people who are suicidal, significantly decreased levels of BDNF in the brain and serum are observed when compared to the healthy population [60]. An excessive production of cortisol also results in reduced neurogenesis (i.e., formation of new neurons and their connections in the brain), which is observed in depressed patients. Altered levels of BDNF in the brain belong to main factors responsible for disturbances of neuroplasticity and neurogenesis [61]. The last hypothesis of depression regards inflammation, according to which, the development of depression is accompanied by increased levels of proinflammatory cytokines, such as interleukin-1β, interleukin-6, and tumor necrosis factor alpha (TNF-α). The inflammatory hypothesis is partially associated with the HPA axis hyperactivity hypothesis since the immune system also plays its role in the development of chronic stress response in humans [1,58]. The literature data indicate that depression is also somehow related to oxidative stress and a low intake of antioxidants, particularly vitamin A, vitamin C, vitamin E, vitamin B6, vitamin B12, folate, zinc, and selenium. An excessive amount of reactive oxygen species (ROSs) is involved in stress response, neuroinflammation, neurodegeneration, disturbances in activity of neurotransmitters, tissue damage, cell death, improper neuroplasticity, and neurogenesis [62]. In view of the above-listed hypothesis of depression, the mechanism of action of omega-3 polyunsaturated fatty acids fits into the general idea of the antidepressant potential [63].

According to the literature data [64,65], one probable molecular pathway by which omega-3 polyunsaturated fatty acids may influence antidepressant properties is through their metabolites. The metabolism of omega-3 polyunsaturated fatty acids proceeds through three principal pathways [65], involving enzymatic routes dependent on lipoxygenase (LOX), cyclooxygenase (COX), and cytochrome P450 [64,65]. In metabolic processes, EPA and DHA are converted to mediators that exhibit anti-inflammatory effects [66,67,68]. COX and LOX enzymes facilitate the conversion of omega-3 polyunsaturated fatty acids into a variety of products, including prostanoids and leukotrienes, as well as monohydroxy and polyhydroxy fatty acids [69,70,71,72]. Concurrently, cytochrome P450 facilitates the conversion of omega-3 polyunsaturated fatty acids into epoxy- and hydroxylated lipid derivatives [64,69]. Both EPA and DHA reduce the chemotaxis of neutrophils and monocytes, inhibit the synthesis of proinflammatory mediators (i.e., interleukin-1β and TNF-α), and inhibit the proliferation of T-cells [73]. It is believed that omega-3 fatty acids competitively diminish the activity of omega-6 fatty acids, which have pro-inflammatory potential [74]. Therefore, people who ingest significantly more omega-6 fatty acids than omega-3 fatty acids may present a state of systemic low-grade inflammation [75]. Omega-3 polyunsaturated fatty acids, by reducing levels of pro-inflammatory cytokines as well as suppressing signaling pathways dependent on inflammatory factors, may, in this indirect mechanism, stimulate the negative feedback of the HPA axis [76,77]. In fact, several authors demonstrated that supplementation with either omega-3 polyunsaturated fatty acids or fish oil may improve the functioning of the HPA axis [78,79], whereas low levels of omega-3 polyunsaturated fatty acids in plasma may be associated with increased concentrations of CRH and cortisol [80,81].

Sufficient intake of omega-3 polyunsaturated fatty acids can reduce oxidative stress in the brain. It has been demonstrated that these essential nutrients elevate the activity of superoxide dismutase, catalase, and glutathione peroxidase, while reducing the activity of myeloperoxidase, levels of malondialdehyde and nitrites/nitrates, as well as inhibiting lipid peroxidation and protein carbonylation [82,83]. Furthermore, it is assumed that the anti-oxidative potential of omega-3 polyunsaturated fatty acids may be a consequence of their effects on COX-2 enzyme or expression of the nuclear factor-erythroid 2-related factor 2 (Nrf2) [84].

Data in the literature indicate that the anti-neurodegenerative activity of omega-3 polyunsaturated fatty acids is attributed to their ability to reduce neuroinflammation and apoptosis, enhance repair of the central nervous system and production of BDNF, stimulate neurogenesis, synaptic plasticity, neuronal proliferation and migration, prevent degeneration of gray and white matter in the brain, and modulate the activity of both telomerase and mammalian target of rapamycin (mTOR) [85,86,87,88,89]. Furthermore, omega-3 polyunsaturated fatty acids have a positive impact on synapses, i.e., synaptic development (formation of new synapses and dendritic spines), synaptic plasticity, and synaptic transmission [90]. Sidhu and colleagues [90] reported a considerable loss of proteins responsible for synaptic plasticity and activity (i.e., fordin-α, synaptopodin, PSD-95, SV2B, SNAP25, SNAP-α, NR2B, AMPA2, and AP2), which was accompanied by cognitive decline in DHA-deficient aging (15 months old) mice. These changes were mitigated by the restoration of DHA brain levels (after feeding animals with a DHA-adequate diet for 2 months).

An additional mechanism being considered by researchers may be the role of omega-3 polyunsaturated fatty acids in maintaining membrane stability and flexibility, which is essential for proper functioning and interactions of receptors, channel proteins, and enzymes that are located in the membranes, neurotransmitter connections, and cell signaling processes [91,92]. The content of DHA within lipid membranes is of significant importance in increasing their fluidity, expansion, and membrane fusion; therefore, this acid plays a considerable role in neurite outgrowth [92,93,94,95,96]. Existing evidence demonstrates that omega-3 polyunsaturated fatty acids are important for the synthesis, synaptic release, and uptake of neurotransmitters associated with the development/successful treatment of depression, i.e., serotonin, dopamine, glutamate, GABA, and norepinephrine. Deficiency in DHA level has been associated with abnormalities in the integrity of neuronal membranes and the signaling of neurotransmitters, including serotonin, dopamine, and norepinephrine [92,97,98]. These disorders may contribute to the development of mood and cognitive dysfunction, which are characteristic of depression [92]. On the other hand, increased elasticity of the lipid bilayer by DHA facilitates binding of GABA and escalates desensitization of its receptors [99]. Omega-3 fatty acids are also crucial for maintaining an appropriate level of neural membrane potential as well as for neural transmission since they influence sodium and potassium ATPase and calcium, sodium, and chloride ion channels [74].

Moreover, omega-3 polyunsaturated fatty acids influence signaling dependent on the cAMP response element-binding protein, calcium/calmodulin-dependent protein kinase type II, and nuclear factor kappa-B (due to their inhibitory activity on the toll-like receptor 4 or interactions with the peroxisome proliferator-activated receptor-γ), i.e., pathways that play a significant role in the development of depressive disorders [100]. Lastly, DHA and EPA can be metabolized into docosahexaenoyl ethanolamide (DHEA) and eicosapentaenoyl ethanolamide (EPEA), respectively [101]. The last two mentioned belong to endocannabinoid-like derivatives that activate endocannabinoid receptors CB1 and CB2. Modulation of CB1 and CB2 receptors is one of the novel therapeutic strategies that are taken into consideration in the treatment of depression [102].

## 4. Data from Clinical Trials

A correlation between the development of depression and intake of polyunsaturated fatty acids has been found in multiple preclinical and clinical studies [100]. It is believed that a higher ratio of polyunsaturated to monounsaturated and saturated fatty acids is positively associated with depression [28]. On the other side, an inverse correlation between DHA level and both the risk and severity of depression was noted by Kotlega and colleagues [103]. The cross-sectional analysis, taking into account 30,976 participants, carried out by Zhang et al. [104], demonstrated a considerable inverse relationship between consumption of EPA and the prevalence of symptoms of depression. The same inverse correlation between depressive symptoms and consumption of mono-, polyunsaturated fatty acids, and omega-3 and omega-6 fatty acids was found in the cross-sectional study (with 25,294 subjects) by Zheng et al. [105]. Similarly, van der Burg et al. [31] suggested that EPA and DHA could be treated as biomarkers of a response to antidepressant treatment since they noted a significant relationship between increased levels of these omega-3 fatty acids and an improvement in depressive symptoms. However, both the meta-analysis of 31 clinical trials published by Deane et al. [106] and the outcomes obtained by da Silva Sabião et al. [28] showed that omega-3 fatty acids have little or no effect on preventing depression. Da Silva Sabião et al. [28], who did not find any association between the intake of omega-3 fatty acids and the development of depression, suggested that the form of the consumed omega-3 fatty acids (i.e., more biologically active ones, like fatty fish and fish oils) may be important. In fact, in a large-scale national study carried out in South Korea, it was demonstrated that people (particularly women) whose consumption of fish is high (≥4 servings per week) are at significantly (26%) lower risk of developing depression than people who eat a fish dish only once a week or less often [107]. Such a conclusion was based on the outcomes obtained from 31,632 subjects. The meta-analysis carried out by Yang et al. [108], taking into account 10 prospective cohort studies with 109,764 participants, including 6672 patients with depression, revealed that both fish consumption or intake of 500 mg of omega-3 fatty acids/day can modestly lower the risk of depression, particularly in the population of females. A similar conclusion related to greater benefits of the EPA-rich and omega-3-rich diet in women was drawn by Zhang et al. [104] and Zheng et al. [105]. According to the researchers [105], estrogens may influence expression and/or functioning of fatty acids or their metabolism to signaling molecules, or they may have a different impact on neuronal growth, synaptic pruning, and the formation of synapses when compared to male sex hormones. Furthermore, immune response and response to stress vary in women and men [105], and, presumably, the incorporation of omega-3 fatty acids into erythrocytes is more effective in the female population than in the male one [109]. Another important factor associated with the preventive activity of omega-3 fatty acids against depression could be age. Surprisingly, Okereke and colleagues [110] found out that a daily omega-3 supplementation (465 mg of EPA and 375 mg of DHA) resulted in an elevated risk of depression in women (but not in men) aged 50 years or older. Similarly, studies carried out by Vyas et al. [111] and the meta-analysis by Yang et al. [112] did not demonstrate that the intake of omega-3 fatty acids is beneficial in the prevention of late-life depression. However, Mengelberg and colleagues [113], who assessed the effects of omega-3 fatty acids (1491 mg of DHA and 351 mg of EPA/day for 12 months) on cognition and well-being in patients between the ages of 60 and 90, noted that ε4 carriers may benefit from such a supplementation in relation to depression and anxiety symptoms. Both depression and carrying the ε4 allele are associated with a higher level of inflammation, and omega-3 polyunsaturated fatty acids can exert anti-inflammatory activity. Similarly, based on results obtained by Madison et al. [114], supplementation with omega-3 fatty acids could be most effective in people subjected to social stress.

On the other side, a positive association has been found between a higher consumption of omega-6 fatty acids and depression in male patients and overweight patients [28]. Furthermore, higher levels of omega-6 fatty acids to omega-3 fatty acids were detected in patients with depressive symptoms [28,31,115]. The meta-analysis of 12 clinical studies with 66,317 participants, including 4173 patients with depression, supported the notion that the lowering of the omega-6 fatty acids/omega-3 fatty acids ratio in a diet could be beneficial in the context of the prevention of depressive disorders [27]. Okubo et al. [116] also demonstrated that higher blood levels of total omega-6 polyunsaturated fatty acids and LA are significantly associated with a higher rate of depressive symptoms in breast cancer survivors. Such a relationship has not been detected for omega-3 fatty acids. It has been revealed that omega-6 fatty acids increase the expression of nuclear factor-κB, which activates the production of pro-inflammatory cytokines (interleukin 6). As a consequence, reduced plasma levels of tryptophan, diminished production of serotonin, and suppressed expression of BDNF are observed [116].

Results of clinical trials and meta-analyses evaluating the antidepressant potential of omega-3 acids are divergent. According to the outcomes of the recent cross-sectional analysis [104], the EPA-enriched diet may significantly contribute to the improvement of depressive symptoms. Meta-analysis by Luo and colleagues [117] revealed that higher doses of omega-3 fatty acids taken in the early period of major depressive disorder therapy could be superior to lower doses. The meta-analysis taking into account nine randomized clinical trials, carried out by Bai et al. [118], revealed that the treatment with omega-3 fatty acids at a dose higher than 1.5 g/day can be beneficial in alleviating depressive symptoms in older patients (≥60 years old). Based on several studies, it can be assumed that higher doses of EPA than DHA may be more effective in prevention and treatment of depression [24,119,120,121]. Most probably, EPA is more neuroactive (e.g., suppresses neuroinflammation more profoundly), even though its level in the brain is definitely lower than the level of DHA [122]. EPA may also produce more pronounced neurotrophic and neuroprotective effects when compared to DHA. Based on the results by Freud and others [123], EPA’s passage through the blood–brain barrier may be swifter than the one observed for DHA. McNamara et al. [124] suggested that an intake of 1000–1500 mg of DHA per day with a 2:1 EPA:DHA ratio may be optimal in the treatment of patients with established affective disorders. The meta-analysis by Liao and colleagues [125], which included 26 studies with 2160 participants, supported the findings that omega-3 fatty acids are beneficial in depressive patients. The authors demonstrated that pure EPA and formulations of omega-3 fatty acids containing at least 60% of EPA when the dosage of EPA is below 1 g/day are clinically effective. This is in line with other publications, according to which supplements containing EPA at concentrations >50–80% seem to be more effective than those that have a greater amount of DHA when compared to EPA (i.e., >50–80%), and that the most preferable EPA:DHA ratio is 2:1 or 3:1 [104,119,120,126]. Outcomes of the recent meta-analysis by Kelaiditis et al. [127] indicated the antidepressant activity of EPA when it is used at doses within the range of ≥1 g and <2 g/day and when its amount in a formulation containing both EPA and DHA is ≥60%. The authors also demonstrated that EPA given at doses ≥2 g/day did not produce a significant therapeutic effect. This U-shape relationship was in line with results obtained by Sánchez-Villegas and colleagues [128], who reported that a moderate (but not a high) intake of fatty fish and long-chain omega-3 fatty acids may prevent the development of depression. Also, according to the meta-analysis by Wolter et al. [129], supplementation with lower dosages of EPA (≤1 g/day) seems to be more beneficial in improving depressive symptomatology. These results were confirmed by the latest meta-analysis by Chang and others [130]. The authors suggested that a saturation threshold may exist, which could explain why higher doses of EPA do not provide any additional improvement. On the other hand, supplementation with pure DHA or formulations of omega-3 acids containing a higher percentage of DHA than EPA does not produce such benefits. Interestingly, Mischoulon et al. [131] demonstrated that lower doses of DHA (i.e., 1 g/day) could be more effective than the higher ones (2 or 4 g/day) in patients with depression. However, this phenomenon is difficult to explain. Some biological mechanisms related to the absorption of DHA could be responsible for the observed counterproductive effect. Furthermore, consumption of fatty acid emulsions may be more effective than ingestion of capsules thanks to better absorption [132,133]. Therefore, proportions, dosage, and the pharmaceutical formulations of omega-3 acids should be taken into consideration when recommending these preparations to patients with depression. Notably, according to Bayes and colleagues [18], omega-3 polyunsaturated fatty acids appear to have a less pronounced treatment effect on depressive symptoms in men when compared to female patients.

However, some authors claim that omega-3 fatty acids have a very mild effect on the course of depression, and only a small percentage of patients benefit from the consumption of omega-3 fatty acids as compared to untreated depression [134]. According to the Cochrane systematic review from 2021 [135], based on 35 studies with 1964 participants, there is no sufficient evidence supporting the effect of omega-3 fatty acids in major depressive disorder in adults. Only a small-to-modest non-clinically significant effect in alleviating depressive symptoms was detected. The recent meta-analysis by Chang et al. [130] also demonstrated only a subtle, positive influence of omega-3 fatty acids on symptoms of depression in elderly people. Interestingly, a significant improvement was detected after supplementation with DHA. According to the authors, DHA may be more effective than EPA in the reconstruction of the brain structure, that is why the intake of this nutrient is able to alleviate depressive symptoms in patients suffering from dementia. Similarly, due to a high heterogenicity in outcomes, Thakur and colleagues [136] found it difficult to determine the clinical used of omega-3 fatty acids in depression in children and adolescents. Most likely, the administration of omega-3 fatty acids as a monotherapy in depression is not enough to treat this disease, but these nutrients can serve as adjuvants to standard antidepressants [126,137]. The type and dosage of antidepressant therapy seem to be important, since antidepressant drugs have diverse mechanisms of action that can interfere (positively or negatively) with the effects of fatty acids. For example, supplementation with omega-3 fatty acids (i.e., DHA and EPA), given as an adjunctive therapy, can improve residual depressive symptoms in patients with bipolar disorder, which was detected in the meta-analysis of eight double-blind randomized placebo-controlled trials, carried out by Kishi et al. [138]. On the other hand, the meta-analysis carried out by Chambergo-Michilot and colleagues [139] did not give any evidence supporting the recommendation of omega-3 supplementation in patients who are depressed receiving sertraline. Based on the results of three randomized clinical trials, the authors did not detect any significant differences in the reduction of the Beck Depression Inventory scores between the tested groups taking sertraline + omega-3 fatty acids and the control groups on sertraline therapy only. Similarly, individual characteristics of a given patient may be important—it has been demonstrated that patients with higher levels of inflammatory biomarkers (e.g., people with interferon-α-induced depression) may be more prone to respond positively to supplementation with omega-3 fatty acids [140,141,142].

Recently, Wu and colleagues [143] suggested that omega-3 polyunsaturated fatty acids could be a promising dietary intervention in overweight patients with major depressive disorder, since this group of nutrients exerts positive effects on satiety, energy intake, weight, waist circumference, fat mass, and mood. Both obesity and the development of depression are known to be at least partially associated with adipocyte-derived hormones and inflammation. However, in studies carried out by Keshavarz et al. [144], after the daily intake of 1080 mg of EPA and 720 mg of DHA for 12 weeks, female patients with co-morbidity of depression and overweight/obesity did report reduced depression scores and body weight, but the supplementation did not prevent the weight regain in the short-time follow-up, i.e., 1 month after the end of the study.

Wolters et al. [129] noticed that the treatment duration (shorter versus longer) does not significantly influence the effectiveness of omega-3 fatty acids in depressive disorders. The authors explained that, most likely, the differences in the mechanism of action are responsible for this phenomenon, i.e., effects on cytokines, synaptic remodeling, and/or neurogenesis may contribute to the relatively quick responses, whereas incorporation of mega-3 fatty acids into cell membranes in the brain may be associated with responses observed after a longer time.

Several years ago, Guu and colleagues [121] published a practical guideline on the supplementation of omega-3 polyunsaturated fatty acids in the management of major depressive disorder. Based on the outcomes of the literature review, online clinical questionnaire, and online consensus meeting, the authors stated that for adult patients in the acute stage of major depressive disorders, omega-3 polyunsaturated fatty acids should be recommended as an adjunctive treatment to conventional drugs (either concomitantly with the antidepressive drug or when the antidepressant is not effective enough in order to augment its activity) rather than as monotherapy, with a dose of EPA of 1–2 g/day (given either as a pure EPA supplement or as a combination of EPA + DHA; the EPA:DHA ratio should be at least 2:1) for a minimum of 8 weeks. Similarly, in guidelines provided by Sarris and colleagues [145], omega-3 fatty acids (at a dose calculated as 1–2 g of EPA/day, or potentially, in the case of patients with elevated inflammatory markers, up to 4 g/day) are recommended as an adjunctive treatment, not as monotherapy. Even though there is no sufficient data at the moment, according to Guu et al. [121], patients with depression could also benefit from omega-3 polyunsaturated fatty acids given as a longer-term maintenance therapy.

As for children and adolescents with major depressive disorder, similar preliminary clinical guidelines were developed by Chang and Su [48], who recommended that children between 6 and 12 years old should be given omega-3 fatty acids at a dose of 1 g/day, and children under 12 should receive at least 2 g/day. Omega-3 fatty acids should be taken as a combination of DHA and EPA, with a DHA:EPA ratio of 1:2. Such a treatment should last 12–16 weeks. The authors supported the supplementation of omega-3 as an adjunct therapy instead of omega-3 monotherapy when the prescribed antidepressant treatment produced good clinical effect. They also advise checking fasting glucose, lipid profile, and hemogram regularly (every 6–12 months) in children and adolescents who are taking EPA and DHA supplements.

Generally, discrepancies between outcomes of studies may arise from differences between fish consumption in patients (for example, Japanese people eat significantly more fish than the population of Western countries), treatment regimen, the fact that the tested supplement contains only EPA/DHA or a mixture of them, and the proportion of EPA to DHA [146]. The results of the most important studies that were carried out during the last 5 years evaluating effects of the intake of omega-3 fatty acids on depression are presented in Table 3. It should also be mentioned that all research teams found the supplementation with omega-3 very safe and tolerable, with a low incidence of side effects.

The outcomes of clinical trials evaluating a possible association between postpartum depression and intake of polysaturated fatty acids are also inconclusive. For example, several authors reported that either the low intake of omega-3 polyunsaturated fatty acids [162] or a high omega-6 to omega-3 ratio (i.e., above 9:1) [163] may lead to the development of postpartum depression. On the other hand, Nishi et al. [164] demonstrated that EPA may act as an antidepressant in pregnant women, and Harauma et al. [165] showed that consumption of ALA during pregnancy may stabilize the mood in the postpartum period (at 1 month after childbirth). The findings were supported by the meta-analysis carried out by Zhang and colleagues [166], who, based on the outcomes of eight randomized clinical trials involving 638 participants, concluded that omega-3 fatty acids had a significant effect on patients with perinatal (pregnant or postpartum) mild-to-moderate depression, particularly when the EPA:DHA ratio is at least 1.5. Such a treatment seemed to be well-tolerated by women, and it was not related to serious adverse effects. Similarly, Masot et al. [167], based on the outcomes of 14 clinical studies, suggested that DHA could play an important role in the prevention of depression during the gestation period, though the beneficial effect of this omega-3 fatty acid subsides in the postpartum period. On the other hand, Hulkkonen et al. [168] showed only a moderate impact of supplementation with fish oil capsules (containing DHA and EPA) with or without probiotics on prenatal and postnatal depressive symptoms in overweight and obese women. These observations were also confirmed by the outcomes of the recent meta-analyses, which were carried out by Mocking and colleagues [169] and Suradom and colleagues [170]. Mocking et al. [169] advised against standard prescribing omega-3 fatty acids during pregnancy in order to prevent or treat depression (due to the lack of significant effect), but they encourage supplementation of omega-3 fatty acids as an adjunctive treatment in postpartum depression. In turn, Firouzabadi et al. [171], based on their recent findings, suggested that the intake of omega-3 fatty acids during pregnancy may be favorable in relation to postpartum depression. According to de Sousa and dos Santos [172], antenatal supplementation with DHA (1440 mg/day) and EPA (260 mg/day) did not exert any significant effect on depressive symptoms in women over time (from pregnancy until 6 months postpartum) as compared to the placebo-receiving control. Taking into account the above-mentioned facts, it seems that there is a need for further studies investigating the optimal omega-3 fatty acids regimen for depressive symptoms in pregnant women and mothers of newborns that will be carried out at geographically distinct study sites with the participation of women with diverse characteristics [173].

The data from the literature suggest that omega-3 polyunsaturated fatty acids may play a role in physiological changes observed in the menopausal transition, including the development of peri- and postmenopausal depression. The outcomes of some studies provide evidence that the intake of omega-3 fatty acids may at least partially alleviate depressive symptoms observed during menopause [174]. However, the recent systemic review by Iqbal et al. [175], taking into account four clinical trials evaluating effects of omega-3 polyunsaturated fatty acids on menopausal depression, indicated that the findings are inconclusive. Two double-blind clinical studies out of four blinded clinical trials did not demonstrate a reduction in clinical scores. In one trial, the administration of 350 mg of EPA and 50 mg of DHA three times per day for 8 weeks improved the mood of treated female patients. Similarly, when the omega-3 polyunsaturated fatty acid supplementation (1 g/day) was added to citalopram (20 mg/day), after 4 weeks of such a combined intervention, the depression scores in post-menopausal women measured by the Beck’s Depression Inventory scale were significantly lower than the ones obtained for the control group.

Supplementation of omega-3 polyunsaturated fatty acids is generally considered to be safe and well tolerated by patients, including special populations (i.e., children, adolescents, elderly people, pregnant and breastfeeding women) [166,176,177,178]. Long-term observations are scarce, but according to studies by Tatsuno et al. [179], a 52-week supplementation of an omega-3 preparation (TAK-085) that contained 4 g of eicosapentaenoic acid-ethyl ester + docosahexaenoic acid-ethyl ester (465 mg of EPA + 375 mg of DHA as ethyl esters per g) was well-tolerated by participants. Although no serious adverse reactions are linked to omega-3 fatty acids intake, several authors reported that this group of nutrients, particularly when taken at high doses, can cause mild gastrointestinal symptoms (e.g., nausea, belching, fishy taste, and hiccups), mild skin problems (e.g., itchiness, rash, or eruption), or clinically non-significant changes in laboratory parameters (e.g., elevated fasting blood sugar, blood urea nitrogen, low-density lipoprotein cholesterol, or lower levels of hemoglobin and hematocrit) [121]. Thus, the monitoring of potential side effects is recommended when a patient is prescribed omega-3 supplements at higher concentrations [180]. Historical assumptions that omega-3 polyunsaturated fatty acids may influence platelet functions in humans have not been confirmed [181]; therefore, they can be safely taken (at least up to 4 g/day) by patients who are treated with antiplatelet or anticoagulant drugs or people with fibrinogen issues [121].

## 5. Conclusions

Based on the outcomes of clinical studies, systematic reviews, and meta-analyses, as well as the available guidelines on the supplementation of omega-3 fatty acids in depression, it can be concluded that the impact of omega-3 intake on the course of depression can be varied. Whereas some authors highlight the clinical effectiveness of these nutrients, others give evidence that such a therapy has no health benefits, at least in relation to the patient’s mood. Furthermore, the statistical significance of the outcomes does not always mean the clinical significance for a given patient. Recently, a lot of attention has been paid to personalized and integrated interventions in the context of depression. Such an approach encompasses not only evidence-based treatment but also the patient’s unique needs, preferences, and symptoms [182]. Thus, it should be kept in mind that depression is a heterogenous disease with multiple risk factors (psychological, biological, and social) that can be involved in its pathogenesis. Moreover, it may be associated with different comorbidities that can influence the effect of pharmacotherapy and the patient’s overall clinical state, even though the meta-analysis by Wolters and colleagues [129] did not demonstrate any differences in the effectiveness of omega-3 fatty acids between subgroups with various co-morbidity statuses and severity of depression. The dietary status of a depressed person, their age, metabolic functions, and even gender may have an impact on both the absorption and effectiveness of omega-3 fatty acids. Polymorphisms in the fatty acid desaturase gene cluster may also influence tissue levels of omega-3 fatty acids [183]. Most probably, not all depressed people require the supplementation of omega-3 polyunsaturated fatty acids to fight their disease, and this is why not all depressed people benefit from an increased intake of EPA and/or DHA. Similarly, prevention of the development of depression is not so simple as to be limited to the supplementation of a sufficient amount of omega-3 fatty acids. However, some subpopulations of patients with depression, i.e., children, elderly people, postmenopausal women, women with perinatal depression, patients with depression associated with (low-grade) inflammation, dietary deficiency, or overweight/obesity, may be particularly responsive to the treatment with omega-3 fatty acids. Furthermore, such an approach may be specifically helpful when it is co-administered with conventional antidepressant therapy.

It should be noted that clinical trials that evaluated the effectiveness of the antidepressant potential of omega-3 fatty acids had limitations. First of all, an extreme heterogeneity in study design has been observed. In fact, most of the cited trials were randomized, double-blind, and controlled, but inclusion/exclusion criteria, diagnostic criteria (*The Diagnostic and Statistical Manual of Mental Disorders* or *the International Classification of Diseases*), depression assessment scales (i.e., the Hamilton Depression Scale, Beck Depression Inventory, Children’s Depression Rating Scale-Revised, Montgomery– Åsberg Depression Rating Scale, and others), and age groups (younger children with or without adolescents, adults without a subgroup analysis based on age) were different. In several studies, patients also took antidepressants or were subjected to psychotherapy, whereas in other trials, patients were given only the supplementation of omega-3 fatty acids. Usually there was no information about the previous antidepressant treatment, time of taking the supplement (fatty food improves absorption of omega-3), background diet, potential other sources of omega fatty acids consumed by participants (i.e., with food), or the baseline omega-3 intake. The baseline omega-3 fatty acid intake might influence the effectiveness of the additional supplementation of these nutrients. Moreover, different treatment schedules, EPA and/or DHA doses, EPA:DHA ratios, and other active substances as additional ingredients of omega-3 supplements were used (i.e., vitamin E). For example, a lower risk of depression associated with increased consumption of fish may not be solely attributed to omega-3 fatty acids but to vitamin D, vitamins B (B2, B6, and B12), selenium, or folate, which are also found in fish and have an antidepressant potential [184,185,186,187]. Therefore, in our opinion, future research should enroll larger cohorts, be more uniform in relation to study design and patient characteristics (including severity of depression, comorbidities, dietary status, baseline levels of omega-3 fatty acids, BMI, and sex), have extended follow-up periods (focused on both clinical effectiveness and safety profile), and assess patient compliance. It would be beneficial to check the nature of the EPA-DHA interaction, whether they act synergistically, and why combinations with higher EPA (than DHA) concentrations have a better effect on people with depression. Similarly, further research on potential interactions between antidepressant drugs and omega-3 fatty acids are required in order to check their molecular mechanism and investigate which group/groups of antidepressants would act the most effectively when co-administered with EPA and/or DHA. Antidepressant drugs have different mechanisms of action, which depend on their class, and some of them may alter the effects of omega-3 fatty acids.

Even though the clinical effectiveness of omega-3 fatty acids in depression is uncertain, such a supplementation has a positive safety profile with a low risk of adverse reactions in diverse populations, including vulnerable subgroups (i.e., children, elderly people, and pregnant women). Therefore, prescribing the use of EPA and/or DHA to a depressed patient, particularly as an adjuvant treatment, is relatively safe. The quality of recommended omega-3 supplements is of the utmost importance, as is their proper storage. They are crucial in terms of bioavailability and pharmacodynamics. Omega-3 fatty acids are known to be sensitive to oxidation and rancidity. Therefore, they should be protected from oxygen, heat, and light [188,189]. It is recommended to check the quality of omega-3 supplements in non-responsive patients.

To sum up, although with mixed results, the available literature data give some evidence for the utility of omega-3 fatty acids in the management of certain types of depression. In our opinion, for sure, supplementation with omega-3 fatty acids should not be treated as an alternative to antidepressant drugs, but it can serve as a promising, safe adjuvant treatment. However, definite conclusions are difficult to draw; prudence is recommended, and more studies in this field are needed.

## Figures and Tables

**Table 2:** Content of ALA, EPA, and DHA in selected food sources [47].

Food		Measure	g/Measure		
			ALA	EPA	DHA
Egg	yolk (dried)	1 cup			0.170
	whole (dried)				0.169
	whole (cooked, hard-boiled)			0.007	0.052
Fish	mackerel (salted)	1.0 piece		1.295	2.372
	salmon	1.0 filet		0.583–0.977	1.246–1.642
	herring			1.300–1.788	1.272–1.580
	mackerel			0.429–1.006	0.615–1.569
	bluefish			0.378	0.778–0.779
	trout			0.161–0.669	0.408–0.744
	sea bass			0.208	0.560–0.562
	sucker			0.303	0.460
	pompano			0.197	0.444
	tilefish	0.5 filet		0.258	1.100
	wolffish			0.468	0.482
Fish oil	salmon	1.0 tbsp		1.771	2.480
	sardine			1.379	1.449
	menhaden			1.791	1.164
	herring			0.853	0.572
	cod liver			0.310	0.494
Meat	lamb	3.0 oz		0.053–0.139	0.383–1.088
	beef			0.087–0.130	0.570
	pork				0.391
	turkey	1.0 breast		0.017–0.082	0.199
	chicken	1.0 cup		0.022–0.042	0.112–0.154
Mollusks		3.0 oz		0.033–0.300	0.173–0.430
Nuts	walnuts (black, dried)	1.0 cup	3.346		
	mixed nuts (dry roasted, with peanuts and salt)		2.662		
	pistachio nuts (dry roasted, with or without salt)		0.261		
	pine nuts (dried)		0.151		
	peanuts (all types, dry-roasted, without salt)		0.037		
	Brazil nuts (dried, unblanched)		0.024		
Oil	flaxseed (cold pressed)	1.0 tbsp	7.258		
	flaxseed (contains added sliced flaxseed)		6.703		
	canola		1.279		
	soy (industrial, refined, for woks and light frying)		0.940		
Seeds	chia seeds (dried)	1.0 oz	5.055		
	hemp seed (hulled)	3.0 tbsp	2.605		
	beans (navy, mature, raw)	1.0 cup	1.119		
	beans (cooked, boiled, with salt)		0.375		
	beans (cooked, boiled, without salt)		0.322		
	pumpkin and squash seed kernels (dried)		0.155		
	pumpkin and squash seed kernels (roasted, with or without salt)		0.131		
	sunflower seed kernels (oil roasted, with or without salt)		0.103		
Shrimp		3.0 oz		0.093–0.115	0.105–0.120

ALA, alpha-linolenic acid; DHA, docosahexaenoic acid; EPA, eicosapentaenoic acid.

**Table 3:** Omega-3 fatty acid intake and its effects on depression: recent studies.

Type of Depression	Type of Study	Participants	Intervention	Main Outcomes	Ref.
Major depressive disorder	Randomized, double-blind, placebo-controlled trial	Adolescents (12–19 years old), 51 participants	Intervention group: Capsules containing EPA + DHA in a 2:1 ratio taken for 10 weeks. Each participant started with an initial dose of 1.2 g/day. Doses were raised in increments of 0.6 g/day every 2 weeks (maximum possible dose of EPA + DHA was 3.6 g/day, i.e., 2.4 g/day + 1.2 g/day, respectively). Control group: Placebo capsules (corn and soybean oils) taken for 10 weeks.	Supplementation of omega-3 fatty acids was not superior to placebo in reducing depression severity as measured by the Children’s Depression Rating Scale-Revised or the Beck Depression Inventory-II.	[147]
Major depressive disorder	Randomized, double-blind, placebo-controlled clinical trial	Adults, 50 participants	All participants received sertraline (50–200 mg/day). Intervention group: 1 capsule containing 1000 mg of omega-3-polyunsaturated fatty acids taken for 12 weeks. Control group: 1 capsule containing placebo taken for 12 weeks.	Adjuvant treatment with omega-3 polyunsaturated fatty acids improved symptoms of depression (measured by the Beck Depression Inventory and Montgomery–Asberg Depression Rating Scale), sleep disturbances, anxiety, intolerance of uncertainty, regulation of emotions.	[148]
Major depressive disorder and chronic heart failure	Randomized, double-blind, placebo-controlled trial	Adults, 108 participants	Intervention groups: 1st group received 4 capsules/day of EPA/DHA at a dose of 500 mg per capsule (EPA/DHA ratio of 2:1) for 12 weeks; 2nd group received 4 capsules/day of almost pure EPA at a dose of 500 mg per capsule for 12 weeks. Control group: 4 capsules/day of placebo (corn oil) taken for 12 weeks. Patients not tolerating 4 capsules of the product took a reduced dose (1 capsule was the minimum dose which allowed to stay in the study).	Supplementation with EPA + DHA or EPA was effective in increasing levels of omega-3 fatty acids in erythrocytes, and the omega-3 index compared to placebo. However, no between-group differences in depression symptoms were observed when measured by the Hamilton Depression Scale and the Beck Depression Inventory-II.	[149]
Depression with overweight or obesity	Randomized, double-blind, controlled trial	Adult women, 45 participants	Intervention group: 6 capsules of omega-3/day (each capsule containing 180 mg of EPA + 120 mg of DHA) taken for 12 weeks. Control group: 6 capsules of placebo/day taken for 12 weeks.	Supplementation of omega-3 fatty acids significantly reduced depression versus placebo when measured by the Beck Depression Inventory.	[144]
Mild to moderate depression	Randomized, double-blind, controlled trial	Adults, 90 participants	All participants received psychoeducation. Intervention group: 15 capsules/day containing 300 mg of omega-3 fatty acids (i.e., 558 mg of DHA and 1064 mg of EPA/day) taken for 12 weeks. Control group: 15 capsules/day containing placebo taken for 12 weeks.	A combined intervention of omega-3 polyunsaturated fatty acids and psychoeducation can successfully ameliorate symptoms in people with mild to moderate depression when measured by the Beck Depression Inventory-II. However, the combination of omega-3 polyunsaturated fatty acids and psychoeducation was not better than the psychoeducation intervention alone.	[134]
Major depressive disorder	Randomized, double-blind, controlled trial	Adults, 88 participants	All participants took 4 identical capsules per day for 12 weeks. Each capsule contained either concentrated EPA (750 mg) or DHA (350 mg). Compared groups: 1st group took 4 capsules with EPA/day (3.0 g of EPA/day); 2nd group took 4 capsules with DHA/day (1.4 g of DHA/day); 3rd group took 2 capsules with EPA plus 2 capsules with DHA (1.5 g and 0.7 g of EPA and DHA/day, respectively).	Monotherapy with EPA or the combination of a higher dose of EPA and a lower dose of DHA resulted in a significantly higher remission rate than monotherapy with DHA, but no differences were observed between treatment with EPA versus EPA + DHA when measured by the Kaplan-Meier estimates of cumulative remission rates. Eicosapentaenoylethanolamide levels in plasma were positively correlated with rates of clinical remission.	[150]
Major depressive disorder with or at high risk for coronary heart disease	Randomized, double-blind, controlled trial	Adults, 144 participants	All patients received 50 mg/day of sertraline. Intervention group: 4 capsules with EPA/day (i.e., 2 g of EPA/day) for 10 weeks. Control group: 4 capsules of placebo (corn oil)/day for 10 weeks.	There were no differences between the groups in depression symptoms when measured by the Beck Depression Inventory II and the Hamilton Rating Scale for Depression. There were also no differences when compared the 10-week remission.	[151]
Depressive disorder or mixed anxiety and depressive disorder	Randomized, double-blind, controlled trial	Children (7–18 years old), 58 participants	All patients received standard antidepressant therapy. Intervention group: 20 mL of omega-3 fatty acid-rich fish oil emulsion (providing 2400 mg of total omega-3 fatty acids: 1000 mg of EPA and 750 mg of DHA, EPA:DHA ratio = 1.33:1) taken for 12 weeks. Control group: 20 mL of omega-6 fatty acid-rich sunflower oil emulsion taken for 12 weeks.	Improvement of symptoms measured by the Children’s Depression Inventory (CDI) accompanied by an increase in large HDL subfractions and reduction in small HDL subfractions in the group supplemented with omega-3 fatty acids was observed. A negative correlation between CDI score and HDL-cholesterol and the large HDL subfraction, but not LDL-cholesterol subfractions was detected. CDI score was not associated with erythrocyte membrane fluidity.	[152]
Major depressive disorder or depressive disorder not otherwise specified	Randomized, double-blind, controlled trial	Adolescents (9–21 years old), 42 participants	Intervention group: 3 capsules/day with fish oil taken for 12 weeks (1 capsule contains 450 mg of EPA, 40 mg DHA, and 260 mg of DHA; the total daily dose of EPA + DHA was 2130 mg; EPA/DHA ratio was 1.7:1). Control group: 3 capsules of placebo/day taken for 12 weeks.	Monotherapy with fish oil was not superior to placebo in reducing depressive symptoms in high-risk youths when measured by the Childhood Depression Rating Scale-Revised.	[153]
Major depressive disorder comorbid with cardiovascular diseases	Randomized, double-blind, controlled trial	Adults, 59 participants	Intervention group: omega-3 polyunsaturated fatty acids (2 g of EPA/day and 1 g of DHA/day) taken for 12 weeks. Control group: placebo taken for 12 weeks.	Omega-3 polyunsaturated fatty acids showed efficacy in improving core depression symptoms only in patients with severe major depressive disorder, measured by the Hamilton Depression Rating Scale.	[154]
Depressive disorder (*n* = 31) or mixed anxiety and depressive disorder (*n* = 29)	Randomized, double-blind, controlled trial	Children (7–18 years old), 60 participants	All participants received standard antidepressant therapy. Intervention group: omega-3 fish oil emulsion providing 2400 mg of total omega-3 fatty acids (1000 mg of EPA and 750 mg of DHA, EPA:DHA ratio = 1.33:1) taken for 12 weeks. Control group: omega-6 sunflower oil emulsion containing 2467 mg of omega-6 linoleic acid in triacylglycerol form taken for 12 weeks.	Significant reduction in CDI scores in the group receiving omega-3 fish oil emulsion when compared to the group receiving omega-6 fish oil emulsion. At the baseline, significantly lower concentrations of EPA and DHA levels as well as a higher omega-6/omega-3 ratio were detected in depressed patients.	[155]
Major depressive disorder	Randomized, double-blind, controlled trial	Adults, 61 participants	Intervention groups: 1st group took 1 g of EPA/day for 12 weeks; 2nd group took 2 g of EPA/day for 12 weeks; 3rd group took 4 g of EPA/day for 12 weeks. Control group: placebo taken for 12 weeks.	4 g of EPA reduced depression symptoms measured by the Depressive Symptomatology, Clinician-Rated version.	[156]
Major depressive disorder	Randomized, double-blind, controlled trial	Adults, 45 participants	Intervention groups: EPA in the form of capsules containing approximately 590 mg of EPA and 152 mg of DHA. 1st group: 1 g of EPA/day taken for 12 weeks; 2nd group: 2 g of EPA/day taken for 12 weeks; 3rd group: 4 g of EPA/day taken for 12 weeks. Control group: placebo taken for 12 weeks.	EPA given at a dose of 4 g/day reduced clinical symptoms of depression, defined as achieving ≥50% reduction in the Inventory Depressive Symptomatology-30 scores.	[157]
Major depressive disorder or bipolar disorder	Randomized, double-blind, controlled, cross-over study	Adults, 42 participants	All patients received either antidepressant medications (SSRIs, desvenlafaxine, agomelatine, mirtazapine, vortioxetine) or medications for bipolar disorder (lithium, lamotrigine, clonazepam). Each capsule contained 130 mg of DHA and 35 mg of EPA. Patients received either 260 mg/day (2 capsules) or 520 mg/day (4 capsules) of DHA.	No change in residual symptoms of depression was detected, measured by the Hamilton Depression Rating Scale.	[158]
Major depressive disorder	Randomized, double-blind, controlled trial	Adolescents, young adults (15–25 years old), 233 participants	All participants were subjected to 50-min cognitive behavioral case management sessions every 2 weeks. Intervention group: 4 gelatin capsules with marine fish oil/day (providing 840 mg of EPA, 560 mg of DHA, and 5 mg of vitamin E) taken for 12 weeks. Control group: 4 gelatin capsules with paraffin oil taken for 12 weeks.	No significant effect of the fish oil treatment in major depressive disorder was observed when measured by the Quick Inventory of Depressive Symptomatology, Adolescent Version and the Montgomery–Åsberg Depression Rating Scale. Erythrocyte levels of polyunsaturated fatty acids were not associated with depression severity.	[159]
Depressive disorder	Randomized, open-label, controlled trial	Adolescents (12–13 years old), 71 participants	Intervention group: Paxil (paroxetine, 20 mg) + 3 capsules of omega-3-rich fish oil/day (providing 1941 mg of EPA + 759 mg of DHA; EPA:DHA ratio 2.56:1) taken for 12 weeks. Control group: Paxil (paroxetine, 20 mg)/day taken for 12 weeks.	Omega-3 fatty acids given with Paxil were more effective than Paxil alone in reducing depressive symptoms when measured by the Montgomery– Åsberg Depression Rating Scale. The combined treatment also improved cognitive function and memory better than monotherapy.	[160]
Major depressive disorder with or without high baseline high-sensitivity C-reactive protein	Match-mismatch trial, patients and raters were blind to baseline high-sensitivity C-reactive protein status	Adults, 101 participants	All subjects took their regular antidepressant drugs + 4 capsules of the study product/day (i.e., 2.2 g of EPA + 400 mg of DHA + 800 mg of other fatty acids + 10–24 mg of tocopherol-rich extracts) for 8 weeks. Subjects were allowed to take nutraceuticals other than omega-3 fatty acids (e.g., supplements with vitamins, zinc, or magnesium).	Omega-3 supplementation had a greater antidepressant effect in patients with moderately elevated high-sensitivity C-reactive protein compared to patients with lower high-sensitivity C-reactive protein when measured by the 17-item Hamilton Depression Rating Scale.	[161]

CDI, Children’s Depression Inventory; DHA, docosahexaenoic acid; EPA, eicosapentaenoic acid; HDL, high-density lipoprotein; LDL, low-density lipoprotein; SSRI, selective serotonin reuptake inhibitor.

## Data Availability

No new data were created or analyzed in this study. Data sharing is not applicable to this article.
